# Successful surgical rescue of delayed onset diaphragmatic hernia following radiofrequency ablation using a thoracoscopic approach for hepatocellular carcinoma: a case report

**DOI:** 10.1186/s40792-021-01213-8

**Published:** 2021-05-26

**Authors:** Atsushi Morito, Shigeki Nakagawa, Katsunori Imai, Norio Uemura, Hirohisa Okabe, Hiromitsu Hayashi, Yo-ichi Yamashita, Akira Chikamoto, Hideo Baba

**Affiliations:** 1 Department of Gastroenterological Surgery, Graduate School of Life Sciences, Kumamoto University, 1-1-1 Honjo, Kumamoto, 860-0811 Japan; 2 Department of Gastroenterological Surgery, Graduate School of Medical Sciences, Kumamoto University, 1-1-1 Honjo, Chuo-ku, Kumamoto, 860-8556 Japan

**Keywords:** Radiofrequency ablation, Diaphragmatic hernia, Thoracoscopy, Hepatocellular carcinoma

## Abstract

**Background:**

Radiofrequency ablation (RFA) is widely used as a minimally invasive treatment for hepatocellular carcinoma (HCC). RFA has a low risk of complications, especially compared with liver resection. Nevertheless, various complications have been reported after RFA for HCC; however, diaphragmatic hernia (DH) is extremely rare.

**Case presentation:**

A 78-year-old man underwent thoracoscopic RFA for HCC located at the medial segment adjacent to the diaphragm approximately 7 years before being transported to the emergency department due complaints of nausea and abdominal pain. Computed tomography revealed a prolapsed small intestine through a defect in the right diaphragm, and emergency surgery was performed. The cause of diaphragmatic hernia was the scar of RFA. We confirmed that the small intestine had prolapsed into the right diaphragm, and we resected the necrotic small intestine and repaired the right diaphragm. Herein, we report a case of ileal strangulation due to diaphragmatic hernia after thoracoscopic RFA.

**Conclusions:**

Care should be taken when performing thoracoscopic RFA, especially for tumors located on the liver surface adjacent to the diaphragm. Patients should be carefully followed up for possible DH, even after a long postoperative interval.

## Background

Radiofrequency ablation (RFA) is widely performed as a minimally invasive treatment for hepatocellular carcinoma (HCC). RFA carries a low risk of complications, especially compared with liver resection [1–3]. We have previously performed thoracoscopic RFA for tumors located near the diaphragm, which is difficult to puncture when using the percutaneous approach [4].

Complications of RFA can be divided into three categories, including intrahepatic, extrahepatic, and systemic. Intrahepatic complications include injury to the bile duct, portal vein, hepatic artery, or hepatic vein, which result in bleeding, abscess, or biliary fistula. Extrahepatic complications include pleural effusion, pneumothorax, ascites, and abdominal wall injury. Systemic complications include hepatic failure and acute respiratory failure. However, delayed diaphragmatic hernia following RFA is rare [5]. In this article, we present a case of delayed DH after thoracoscopic RFA for HCC, which subsequently caused ileal strangulation.

## Case presentation

**Fig. 1:**
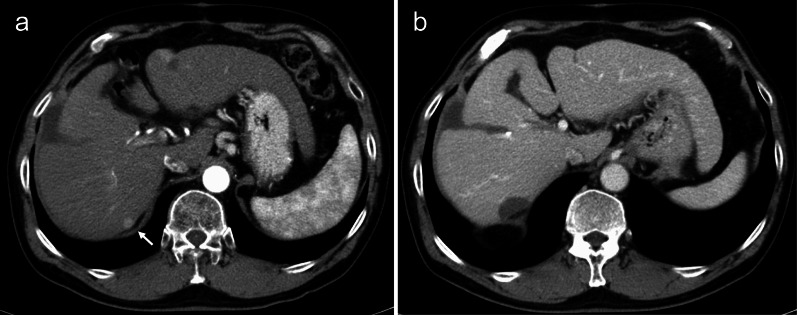
**a** CT image of hepatocellular carcinoma at segment 6/7. It was well enhanced in the arterial phase (arrow). **b** CT image of post-RFA for hepatocellular carcinoma at segment 6/7

**Fig. 2:**
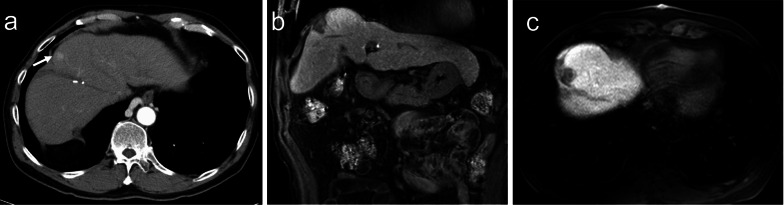
**a** CT image of hepatocellular carcinoma at medical segment. It was well enhanced in the arterial phase (arrow). **b** MRI image of post-RFA for hepatocellular carcinoma at medical segment (coronal view). **c** MRI image of post-RFA for hepatocellular carcinoma at medical segment (axial view)

**Fig. 3:**
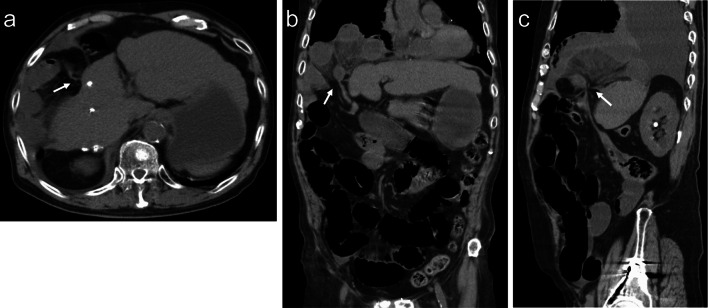
CT image. **a** Axial view, **b** coronal view, **c** sagittal view. The small intestine herniated through the right diaphragm with intestinal expansion (arrow). Pleural effusion was observed in the right chest cavity

A 78-year-old man visited our emergency room with complaints of nausea and abdominal pain. He had previously been treated for HCC and compensated cirrhosis due to chronic hepatitis C. Approximately 10 years ago, the patient underwent a segment 8 partial hepatectomy for HCC. One year later, he underwent RFA for HCC at segment 6/7 with artificial pleural effusion for recurrence (Fig. 1a). Approximately 7 years ago, thoracoscopic RFA was performed for recurrent HCC at the medial segment through the right diaphragm (Fig. 2a). Vital signs at the emergency room were as follows: temperature, 38.2 °C; blood pressure, 149/66 mmHg; pulse rate, 110 beats/min; SpO_2_, 94% (oxygen mask, 4 L/min). A physical examination revealed tenderness in the right upper abdomen; however, rebound tenderness was not recognized. A blood test revealed abnormalities, including a blood urea nitrogen concentration of 63.3 mg/dL, a creatinine concentration of 2.34 mg/dL, a white blood cell count of 24,600/μL, and a C-reactive protein concentration of 9.23 mg/dL. Chest radiograms showed a right opacity and pleural effusion. Computed tomography revealed that the small intestine protruded through the right diaphragm. Intestinal expansion and pleural effusion were also observed (Fig. 3a–c). We diagnosed intestinal obstruction by right DH. As a result of comparison with previous CT images, it was determined that the scar of thoracoscopic RFA at the medial segment caused hernia.

**Fig. 4:**
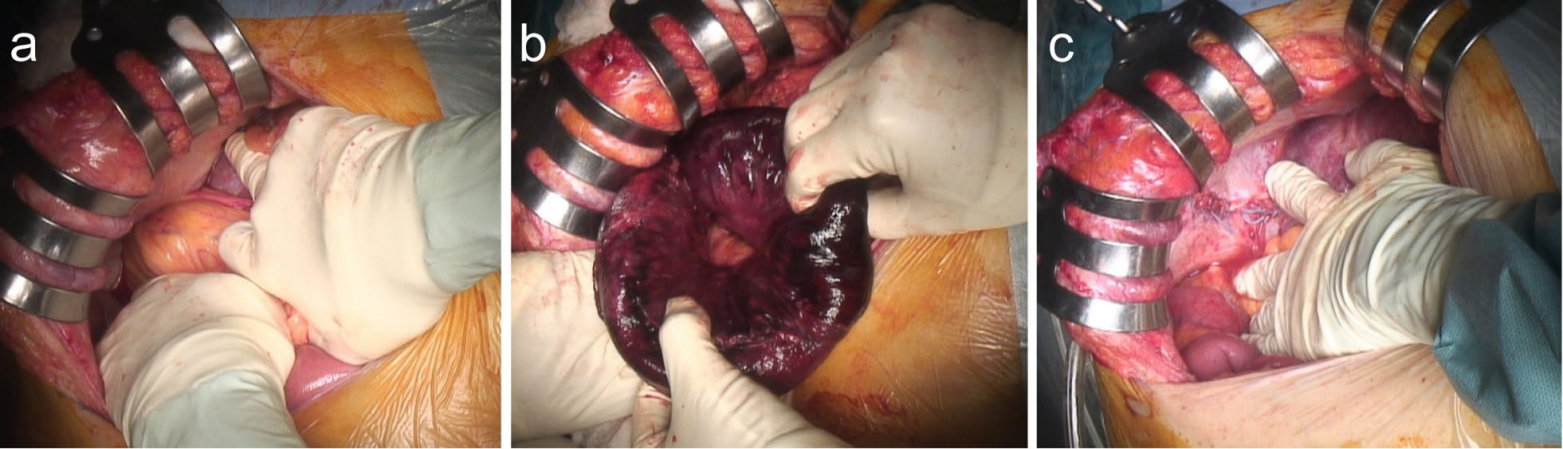
Intraoperative findings. **a** The small intestine herniated into the right diaphragm. **b** The small intestine was necrotic (> 30 cm). **c** After repairing the hole in the diaphragm

Emergency laparotomy was enforced, and we found that the small intestine protruded through the right diaphragm (Fig. 4a), resected the necrotic intestinal tract (Fig. 4b), and repaired the hole in the right diaphragm (8 × 8 cm) using non-absorbable polyester knitting sutures (Fig. 4c). The surgical duration was 246 min, and the volume of blood loss was 215 ml. After surgery, the patient was discharged without any complications on postoperative day 26.

## Discussion

RFA has become the major treatment for HCC, especially HCC with liver cirrhosis. Therefore, it can be speculated that the incidence of rare complications, such as DH as a result of RFA, will inevitably increase.

DH following RFA can be categorized as diaphragmatic injury (DI). Complications within 30 days after RFA are classified as early DI, and complications that occur more than 30 days after RFA are classified as delayed DI [6]. Delayed DI can result in a poorer prognosis compared with early DI. The incidence of death is 30% with delayed DI and 7.1% with early DI [7]. Pekmezci et al. reported that thoracoscopy is an effective tool for diagnosis and subsequent surgical repair of DI [8]. Furthermore, thoracoscopy can eliminate pleural collections, which can cause pyothorax. Therefore, when the diagnosis is uncertain, thoracoscopy is recommended.

The mechanism of DH after RFA has not been clarified. Nagasu et al. reported three causative factors of DH after RFA [9], including the location of the targeted lesions, collateral thermal injury during RFA, and the advanced cirrhosis status had listed. Collateral thermal damage to the diaphragm during RFA to target areas adjacent to the diaphragm is common. In this case, all tumors that were treated with RFA were located adjacent to the diaphragm. Thermal damage to the diaphragm may result in an inflammatory response, leading to fibrosis that could ultimately weaken the muscle fibers of the diaphragm and cause late-onset defects [10]. Poor liver function might prevent the injured tissue from healing adequately, with complications such as ascites and pleural effusion, thereby contributing to further tissue damage [11]. According to past DH reports, most previous patients had a history of RFA treatment for HCCs with right dome lesions of segments 7 or 8 and showed a right-sided DH [12]. This patient had previously undergone S8 resection and had an enlarged medial segment to the right side. It was suggested that this may be the cause of DH at the scar of RFA to medial segment.

**Fig. 5:**
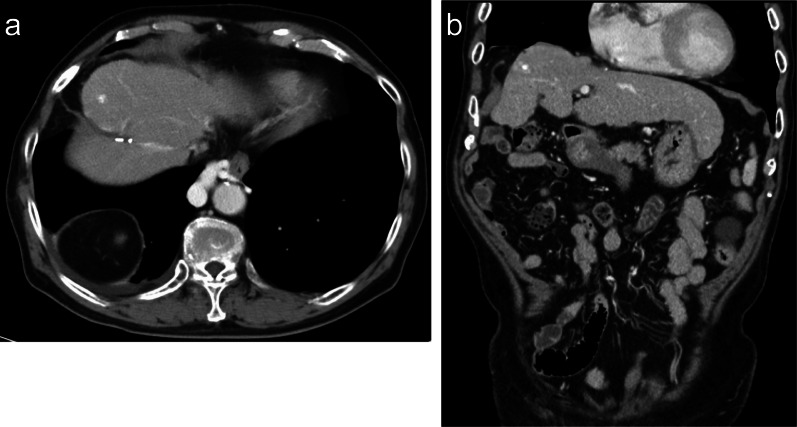
CT image half a year before emergency surgery. **a** Axial view, **b** coronal view. There were no findings of diaphragmatic hernia or Chilaiditi syndrome

Furthermore, Chilaiditi’s syndrome is a condition that is caused by a deterioration in liver function. Moaven et al. reported that the incidence of Chilaiditi’s syndrome inevitably increases in patients with cirrhosis due to liver atrophy or relative atrophy in the medial segment of the left lobe of the liver, which creates space between the diaphragm and liver [13]. Therefore, it is plausible that liver atrophy contributes to perforation and herniation of the diaphragm. In our case, CT 6 months before the onset of DH showed no findings suggestive of Chilaiditi’s syndrome (Fig. 5a, b).

We have previously performed thoracoscopic RFA for HCC under the diaphragm, which is difficult to approach percutaneously [14]. With thoracoscopic RFA, the tumor can be directly confirmed by cutting the diaphragm. We usually cut the diaphragm and puncture the tumor directly using thoracoscopy. The reason we cut the diaphragm is to avoid heat injury. In this case, we did not cut the diaphragm because it was expected that adhesion peeling would be difficult due to repeated treatment.

To avoid damage to the diaphragm, it has been reported that ablation for HCC with artificial pleural effusion or ascites treatment is useful [15, 16]. It will be necessary to consider treatment with pleural effusion or ascites for lesions directly under the dome.

## Conclusion

This case reports DH after thoracoscopic RFA. Care should be exercised when performing thoracoscopic RFA, especially for tumors located on the liver surface just below the diaphragm.

## Data Availability

The datasets supporting the conclusions of this article are included within the article.

## Abbreviations

RFA: Radiofrequency ablation
HCC: Hepatocellular carcinoma
DH: Diaphragmatic hernia
DI: Diaphragmatic injury
